# New insights into the diagnosis of nodular goiter

**DOI:** 10.1186/1756-6614-7-6

**Published:** 2014-06-17

**Authors:** Anhelli Syrenicz, Monika Koziołek, Andrzej Ciechanowicz, Anna Sieradzka, Agnieszka Bińczak-Kuleta, Miłosz Parczewski

**Affiliations:** 1 Department of Endocrinology, Metabolic Diseases and Internal Diseases, Pomeranian Medical University in Szczecin, Szczecin, Poland; 2 Department of Laboratory Diagnostics and Molecular Medicine, Pomeranian Medical University, Szczecin, Poland; 3 Department of Infectious Diseases and Hepatology, Pomeranian Medical University, Szczecin, Poland

**Keywords:** Nodular goiter, Thyroid cancer, Genetic testing

## Abstract

Preoperative diagnostic investigations of nodular goiter are based on two main examinations: ultrasonography of the thyroid gland and ultrasound-guided fine-needle aspiration biopsy. So far, FNAB has been the best method for the differentiation of nodules, but in some cases it fails to produce a conclusive diagnosis. Some of the biopsies do not provide enough material to establish the diagnosis, in some other biopsies cytological picture is inconclusive.

Determining the eligibility of thyroid focal lesions for surgery has been more and more often done with molecular methods. The most common genetic changes leading to the development of thyroid cancer include mutations, translocations and amplifications of genes, disturbances in gene methylation and dysregulation of microRNA. The mutations of *Ras* proto-oncogenes and *BRAF* gene as well as disturbances of DNA methylation in promoter regions of genes regulating cell cycle (e.g. hypermethylation of *RASSF1A* gene and *TIMP-3* gene) play an important role in the process of neoplastic transformation of thyreocyte. The advances in molecular biology made it possible to investigate these genetic disturbances in DNA and/or RNA from peripheral blood, postoperative thyroid tissue material and cytology specimens obtained through fine-needle aspiration biopsy of focal lesions in the thyroid gland.

As it became possible to analyze the mutations and methylation of genes from cell material obtained through fine-needle aspiration biopsy, it would be beneficial to introduce the techniques of molecular biology in the pre-operative diagnosis of nodular goiter as a valuable method, complementary to ultrasonography and FNAB. The knowledge obtained from molecular studies might help to determine the frequency of follow-up investigations in patients with nodular goiter and to select patients potentially at risk of developing thyroid cancer, which would facilitate their qualification for earlier strumectomy.

## Introduction

Nodular goiter is the most common pathology of the thyroid gland. Palpable thyroid nodules are found in 3-7% of adult population and are more frequent in women. Ultrasonography which has been introduced in the diagnosis of thyroid gland has confirmed earlier autopsy reports indicating that focal lesions are found in as many as 50% of clinically normal thyroid glands [1,2]. Preoperative diagnostic investigations of nodular goiter are currently based on two main examinations: ultrasonography of the thyroid gland and ultrasound-guided fine-needle aspiration biopsy. Determining the eligibility of thyroid focal lesions for surgery has been more and more often done with molecular methods providing the information on possible presence of mutations and epigenetic changes which play an important role in malignant transformation [3-12]. Most focal lesions in the thyroid gland are of benign nature. The incidence of thyroid cancer in multinodular goiter is estimated at approx. 5-10%. Thyroid cancer is more common in solitary thyroid nodules (approx. 10-20%) than in multinodular goiter [1,13]. Clinical examination of patients with thyroid nodules remains important component of cancer risk assessment. The risk factors include positive family history (this is especially true for medullary carcinoma and some papillary carcinomas), age (under 20 years and over 60 years), sex (males are at greater risk) and history of head and neck irradiation, particularly in the childhood. Other very important symptoms include dysphonia, raucity and neck pain in patients with hard, not easily movable lump [14,15].

Ultrasonographic examination of focal thyroid lesions, particularly those in multinodular goiter is helpful in selecting one or more foci for fine-needle aspiration biopsy. It is currently believed that the most important criteria for the evaluation of malignant potential of a thyroid nodule is not its size but rather its vascularization, the presence of microcalcifications, height/width ratio, structure (solid or solid-fluid), echogenicity and border margins as well as the presence of so called halo. Hence, thyroid nodules most suspected of malignancy are those with the following ultrasonographic features: enhanced central vascularization or no flow in power doppler; microcalcifications; nodule height exceeding its width; solid lesions are more suspicious than solid-fluid ones; hypoechogenic lesions raise more concerns than isoechogenic ones; lesions with rough margins and those without a halo or lesions with irregular, thick halo are more suspicious [16]. Nowadays, ultrasound-guided fine-needle aspiration biopsy is a gold standard in the diagnosis of nodular goiter. It is technically simple, safe and inexpensive. Cytology assessment of the specimens obtained through fine-needle aspiration biopsy is based on international classification known as the Bethesda System of Reporting Thyroid Cytopathology [17]. According to this classification, the findings of fine-needle aspiration biopsy of thyroid nodule can be divided into 6 groups of diagnostic cytopathology categories: I-non-diagnostic or unsatisfactory, II- benign, III- atypia of undetermined significance or follicular lesion of undetermined significance, IV- follicular neoplasm or suspicious for a follicular neoplasm, V- suspicious for malignancy and VI- malignant. Cytopathology diagnoses falling within groups IV, V and VI are indications for surgery. Diagnoses classified as group III and those classified as group I mean that fine-needle aspiration biopsy should be repeated. It is also worth emphasis that even the diagnosis of a benign lesion (group II ) in fine-needle aspiration biopsy carries 3% risk of false negative result [17]. Irrespective of the chosen classification of cytopathology findings, one should assume that approx. 20% of thyroid nodule biopsies produce results that require final diagnosis based on postoperative histopathology report [13].

The scintigraphy of the thyroid gland is currently considered less useful in the diagnosis of possible malignancy in the nodular goiter since it has been demonstrated that malignant lesions may be found not only in cold nodules but also in approx. 3% of solitary hot thyroid nodules [18].

Thyroid cancer is the most common malignancy of endocrine system accounting for 2.5% of all cancers diagnosed in humans [19]. It has been diagnosed more frequently since 1990s, particularly in women [20]. In Poland, there are 1500 to 1800 new cases of thyroid cancer per year [21]. The most common histological type of thyroid cancer is papillary thyroid carcinoma (PTC) and the second most common differentiated thyroid cancer is follicular thyroid carcinoma (FTC); they account for 80% and 15% of all thyroid cancers, respectively [22,23]. PTC and FTC are both classified as differentiated cancers of the thyroid gland, deriving from highly differentiated follicular cell which is dedifferentiated in the presence of various factors, fails to undergo apoptosis and becomes capable of uncontrolled proliferation and forming metastases [24,25]. Neogenesis is a multistage process, involving multiple genes as well as endo- and exogenous factors. At the initial stage of neogenesis called initiation, the cell is damaged. The second stage called promotion is the clonal expansion of abnormal cell triggered by mitogenic signal, which produces a group of dividing cells with still benign genotype. The third stage is neoplastic transformation of some clonal cells caused by further genetic changes – oncogenesis is part of progression stage.

## Review

The most common genetic changes resulting in the development of thyroid cancer are mutations, translocations and amplifications of genes, disturbances in gene methylation and dysregulation of microRNA [3,4,6,26]. The advances in molecular biology made it possible to investigate these genetic disturbances in DNA and/or RNA from peripheral blood, postoperative thyroid tissue material and cytology specimens obtained through fine-needle aspiration biopsy of focal lesions of the thyroid gland. They cast new light on the genesis of benign and malignant lesions in the thyroid gland and also opened new perspectives for preoperative diagnosis of focal thyroid lesions. The first Polish center to conduct molecular tests of cytology material obtained from fine-needle aspiration biopsy of the thyroid gland was in Szczecin and these tests were aimed at the detection of somatic mutations of TSH receptor gene and G protein alpha chain [27,28]. New molecular diagnostic tools applied to fine-needle aspiration biopsy allowed for more precise qualification of patients for total or partial strumectomy. The key role in the neoplastic transformation of thyroid follicular cell is played by the inactivation of suppression genes and activation of oncogenes [3,4,24,25,29]. Mutations observed in thyroid cancers usually affect RAS, BRAF, PTEN, CTNNB1, TP53, IDH1, ALK and EGFR genes [9,12,25,30].

BRAF gene plays a very important role in the etiopathogenesis of papillary thyroid carcinoma [7,8,31-33]. The predominant BRAF gene mutation reported in PTC, observed in 36-80% of PTC cases is the thymine-to-adenine transversion at position 1799 (T1799A) in exon 15, resulting in the substitution of valine (V) by glutamic acid (E) at codon 600 (V600E) [22,33-40]. This specific V600E BRAF mutation represents 99% of all BRAF mutations found in thyroid cancer [6]. Many studies prove that this mutation is found only in papillary thyroid carcinoma and in few cases of anaplastic cancer [34,36,41]. The presence of BRAF T1799A oncogene is an unfavorable prognostic factor in PTC as it increases the aggressive nature of cancer through raising its invasiveness, accelerating relapses and the occurrence of metastases [23,33,34,36,37,42-44] BRAF gene encodes BRAF protein. BRAF protein belongs to a class of serine/threonine kinases and subfamily of RAF proteins [8,45,46]. Cytoplasmic RAF proteins make up RAS-RAF-MEK-ERK pathway which is involved in the transduction of mitogenic signal from the cell surface to cell nucleus [47-49]. This pathway using tyrosine kinase receptor is a mitogen-activated kinase cascade called MAPK (Mitogen-Activated Protein Kinase) [32,47-49]. BRAF gene mutation activating MAPK pathway is most likely the main contributor to the development and progression of PTC. T1799A BRAF oncogene is present at all stages of PTC progression, it may even be there at early stages of the development of micropapillary cancer [6,31,34,50]. The presence of BRAF mutation in cytology material obtained from FNAB of thyroid nodule indicates the necessity of surgical treatment. It should be remembered, however, that false positive results indicating the presence of BRAF in the nodule are reported in 0.2-5.7% of cases, while false negative results are found in 1.9-5.8% of cases [51-54]. Since in some cases of thyroid nodule biopsy, the specimens for cytology assessment do not contain enough cells with mutated BRAF and the result obtained is negative despite the mutation in thyroid nodule, currently there are indications to re-evaluate BRAF mutation in follow-up biopsy performed a few months later, especially when the nodule presents ultrasonography features of malignancy and cytology assessment of focal thyroid lesion gives non-diagnostic results, there are signs of atypia or the nodule is of benign nature [11].

Following BRAF mutation, the second most common mutation observed in thyroid cancer are the mutations of RAS proto-oncogenes, which play an important role in the initiation of thyreocyte neoplastic transformation [12,55,56]. Proto-oncogenes of RAS family (N-RAS, H-RAS, K-RAS) are located on 1, 11 and 12 chromosome, respectively. They are involved in the control of growth and differentiation of cells. They encode G membrane proteins showing intrinsic GTP-ase activity and participate in signal transmission from membrane tyrosine kinase receptor to cell nucleus using both MAPK and PI3K-AKT pathways. Mutations at codons 12, 13 and 61 transform these proto-oncogenes into active oncogenes [6,57]. Mutations of RAS proto-oncogene make the protein encoded by this proto-oncogene lose its intrinsic GTP-ase activity and there is a constitutive activation of signal transduction pathway. In thyroid tumorigenesis, PI3K-AKT is a preferable pathway [55]. RAS gene mutations are observed both in benign and malignant thyroid neoplasms. These mutations can be found in 40-50% of FTC cases, in 5-20% of PTC cases, in 20-40% of poorly differentiated and anaplastic cases as well as in approx. 30% of follicular adenomas [9,32,35,56,58,59]. RAS-positive follicular adenoma may be a precursor of both follicular cancer and follicular variant of papillary carcinoma [6]. Recent studies have emphasized an important role of RAS mutation as a valuable diagnostic marker in tumors with very difficult or impossible diagnosis based on cytology assessment of fine-needle aspiration biopsy material, which is true for follicular variant of papillary cancer and follicular adenoma [60,61]. Diagnostic difficulties with follicular variant of papillary cancer result from the absence of papillary proliferation and limited nuclear features typical for papillary cancer. To differentiate between adenoma and follicular cancer, the assessment of vascular invasion and capsule infiltration is necessary, which cannot be based on cytology material obtained from fine-needle aspiration biopsy [39,56]. Most RAS-positive thyroid nodules with indeterminate cytology and without suspicious ultrasonography features turn out to be a follicular form of papillary cancer with low level of malignancy in post-operative histopathology examination [12]. RAS mutations are found in a large percentage of poorly differentiated and anaplastic thyroid cancers, therefore, it seems advisable to consider surgical treatment of all RAS-positive thyroid nodules to prevent cancer progression [12].

Yet another kind of genetic changes found in thyroid cancer are oncogenic rearrangements resulting from gene translocations, with RET/PTC and PAX8/PPARγ being the most common [62-64]. More than 10 types of RET/PTC translocation have been described but the two most frequently occurring are RET/PTC1 and RET/PTC3 [62,65]. RET is a proto-oncogene encoding RTK. RET/PTC is formed as a consequence of genetic recombination between the 3’ portion of RET tyrosine kinase and the 5’ portion of a partner gene. In the case of RET/PTC1 rearrangement, the partner gene is CCDC6 known as H4 (coiled-coil domain-containing gene 6), while in RET/PTC3 rearrangement, the partner gene is NCOA4 known as ELE1 (nuclear receptor co-activator 4). The structural basis for RET/PTC transformation is close vicinity of RET and the partner gene in cell nucleus [66,67]. The consequence of this rearrangement is ligand-independent dimerization and constitutive activation of RET tyrosine kinase [25]. RET/PTC1 is the most frequent type accounting for 60-70% of re-arrangements and RET/PTC3 is observed in 20-30% of PTC cases [68]. RET/PTC1 re-arrangements are more common in classic forms of papillary cancer and in papillary microcarcinoma [3,69]. On the other hand, RET/PTC3 re-arrangements are more common in solid papillary cancers, which was observed especially in the Ukraine and Belarus after Chernobyl disaster [70].

Another important re-arrangement observed in thyroid cancer is PAX8/PPARγ as a consequence of the translocation of genetic material between chromosomes 2 and 3. Then, PAX8 gene which encodes thyroid-specific transcription factor domain is combined with PPARγ (peroxisome proliferator-activated receptor-γ). PAX8/PPARγ rearrangement is observed mainly in follicular cancers, but also in the follicular form of papillary cancer and follicular adenomas [64,71].

It is not only the translocation of genetic material but also the amplification of oncogenes that may play a very important role in the thyroid tumorigenesis. This is especially the case in the genes encoding MAPK pathway kinases using tyrosine kinase receptor but also in the genes encoding PI3K-AKT pathway. Elevated number of oncogene copies are more common in anaplastic cancer than in differentiated cancers of the thyroid gland, which suggests that it may be of considerable relevance for cancer aggressiveness and the rate of its progression [72].

It has been demonstrated that neoplastic transformation of the thyroid gland is also affected by epigenetic mechanisms, i.e. the mechanisms influencing the regulation and modification of genetic material, not affecting the nucleotide sequence [4,5,25,29,73-75]. These mechanisms include DNA methylation and histone modification. DNA methylation takes place through covalent modification of cytosines and it is catalyzed by DNA methyltransferases, which attach methyl group at the carbon 5’ position of cytosine ring. This modification applies only to cytosines (C) which are followed by guanine (G) in the sequence. CG sequences are grouped in the genome sites known as CpG islands, where CG dinucleotide repetitions extend over 1,000 - 2,000 base pairs [76]. In the case of genes with vital significance for fundamental cellular processes, with widespread expression in the tissues, CpG islands associated with them are almost always found on 5’ side of encoding sequences, typically in the promoters of these genes. The general mechanism of silencing expression of hypermethylation-dependent DNA genes has several aspects. The most important of them is to prevent the binding of transcription factors to promoters and sequences regulating transcription, on the basis of spatial conflict. These data are consistent with observations which imply that CpG islands of suppressor genes in healthy somatic cells are usually characterized by low levels of methylation or no methylation at all. During oncogenesis, hypermethylation of these sites often occurs, which causes silencing of their expression. Thus, in the selective strategy of a neoplasm, hypermethylation of genes is aimed at marking those genome areas which are to undergo deletion processes, leading to irreversible loss of growth control. The result of this situation is not only accelerated growth of cells but also the beginning of particularly dangerous genetic instability [77,78]. The genes controlling the proliferation of cells, which undergo hypermethylation in the papillary carcinoma include TIMP-3 (tissue inhibitor of metalloproteinase-3, inhibitor of extracellular metalloproteinases), DAPK (calcium-dependent protein kinase), taking part in programmed cell death, SLC5A8 (sodium symporter), DNA repair genes (hMLH1, PCNA) and thyroid-specific genes (NIS-sodium-iodine symporter, TSHR- thyroid stimulating hormone receptor). The genes encoding the suppressors of neoplasia undergoing hypermethylation in follicular cancer include PTEN (phosphatase inhibiting one of mitogen signal transduction pathway), RASSF1A (signal protein of mitogen RAS pathway), thyroid-specific genes (NIS, TSHR) and TRbeta (receptor beta for thyroid hormones) [1,4,5,75,79,80]. Special attention should be paid to TIMP-3 and RASSF1A genes taking part in the tumorigenesis of the thyroid gland [4,5,29]. TIMP-3 inhibits the growth, angiogenesis and invasion of many cancers. Hypermethylation of this gene is particularly important in the onset of papillary thyroid cancer. It has been demonstrated that there is a correlation between loss of TIMP-3 gene function as metalloproteinase inhibitor associated with hypermethylation and extrathyroid invasion of papillary carcinoma, lymph node metastases and multifocal nature of this cancer [4,75,81,82]. Protein products of RASSF1A suppressor gene participate in controlling cell cycle, controlling the differentiation and proliferation of cells through direct regulation of transcription and regulation of proapoptic signal pathways. Epigenetic silencing of this gene expression through promoter hypermethylation may lead to unauthorized divisions of mutated cells. Decreased expression of RASSF1A gene and/or reduced activity of its protein products are also affected by changes in DNA sequence related to the acquisition of genome instability by neoplastic cells, resulting from loss of heterozygosity as well as from the instability of microsatellite sequences [1,4,5,29]. Hypermethylation of RASSF1A gene is observed both in benign thyroid neoplasms and in thyroid cancers, particularly in FTC. Methylation levels above 50% of alleles was only observed in follicular thyroid cancer, while it was not observed in benign neoplasms of the thyroid gland, suggesting that methylation through silencing both RASSF1A gene alleles may play an important role in the pathogenesis and development of follicular thyroid cancer [1,4,5,29].

## Conclusion

New opportunities for the analysis of mutation and methylation of genes obtained from fine-needle aspiration biopsy presented in this article confirm clinical benefits from introducing molecular studies into pre-operative diagnostic investigations of the thyroid gland as a valuable method complementary to ultrasonography and cytology evaluation of thyroid bioptates, particularly when qualifying patients with follicular adenomas and follicular lesions with undetermined significance for surgical treatment.

## Abbreviations

FNAB: Fine-needle aspiration biopsy; Approx: Approximately; PTC: Papillary thyroid carcinoma; FTC: Follicular thyroid carcinoma; RNA: Ribonucleic acid; DNA: Deoxyribonucleic acid; V: Valine; E: Glutamic acid; RET: a proto-oncogene encoding RTK; TSHR: Thyroid stimulating hormone receptor; CCDC6: Coiled-coil domain-containing gene 6; MAPK: Mitogen-activated protein kinase; C: Cytosine; G: Guanine; NCOA4: Nuclear receptor co-activator 4; ELE1: Nuclear receptor co-activator; 4TIMP-3: Tissue inhibitor of metalloproteinase-3; DAPK: Calcium-dependent protein kinase; SLC5A8: Sodium symporter; NIS: Sodium-iodine symporter; TRbeta: Receptor beta for thyroid hormones; RASSF1A: Signal protein of mitogen RAS pathway; PPARγ: Peroxisome proliferator-activated receptor-γ.

## Competing interests

The authors have non-financial competing interests (political, personal, religious, ideological, academic, intellectual, commercial or any other) to declare in relation to this manuscript.

## Authors’ contributions

Prof AS have made substantial contributions to conception and design, acquisition of data and analysis and interpretation of data; have been also involved in drafting the manuscript, have given final approval of the version to be published; MK **-** have made substantial contributions to conception and design, acquisition of data and analysis and interpretation of data; have been also involved in drafting the manuscript, AC - have been also involved in drafting the manuscript, AS - participated in the sequence alignment and drafted the manuscript, ABK - carried out the molecular genetic studies, MP - carried out the molecular genetic studies. All authors read and approved the final manuscript.
